# Comparison of the tidal volume by the recruitment maneuver combined with positive end-expiratory pressure for mechanically ventilated children

**DOI:** 10.1038/s41598-023-45441-4

**Published:** 2023-10-31

**Authors:** Masanori Tsukamoto, Maho Goto, Takashi Hitosugi, Kazuya Matsuo, Takeshi Yokoyama

**Affiliations:** 1 Department of Dental Anesthesiology, Graduate School of Medical and Dental Sciences, Kagoshima University, 8-35-1 Sakuragaoka, Kagoshima, Kagoshima 890-8544 Japan; 2 Department of Dental Anesthesiology, Graduate School of Dental Science, Kyushu University, Fukuoka, Japan; 3 Department of Dental Anesthesiology, Faculty of Dental Science, Kyushu University, Fukuoka, Japan; 4 Department of Control Engineering, Kyushu Institute of Technology, Kitakyushu, Japan

**Keywords:** Medical research, Physics

## Abstract

The recruitment maneuver (RM) combined with PEEP to prevent atelectasis have beneficial effects. However, the change in tidal volume (V_T_) due to RM combined with PEEP in pediatric patients during the induction of general anesthesia is unknown. Therefore, we assessed the effects of RM combined with PEEP on V_T_. Pediatric patients were divided into three groups: infants, preschool children, and school children. The RM was performed by maintaining pressure control continuous mandatory ventilation (PC-CMV) with a 15 cmH_2_O and PEEP increase of 5 cmH_2_O. V_T_, respiratory function and hemodynamics were monitored before and after RM combined with PEEP. V_T_ (mL) /ideal body weight (kg) before vs. after RM combined with PEEP were 9 vs 12 mL/kg (p < 0.05) in the infants, 9 vs 11 mL/kg (p < 0.05) in the preschool children, 8 vs 10 mL/kg (p < 0.05) in the school children, respectively. HR and BP before and after RM combined with PEEP increased by 2–3% and decreased by 4–7% in all groups. RM combined with PEEP resulted in an increase in V_T_ per ideal body weight (1.1–1.2%). Therefore, this RM combined with PEEP method might improve the lung function in pediatric patients.

## Introduction

General anesthesia with tracheal intubation and mechanical ventilation is widely used in patients undergoing a variety of surgical procedures^1–3^. However, alveolar collapse is multifactorial in origin and begins quickly after tracheal intubation^4,5^. Reduced lung compliance^6–8^ and pulmonary gas exchange impairment may result in decreased oxygenation of arterial blood^9–11^. Absorption atelectasis occurs when the flux of oxygen from the alveoli into the capillaries exceeds that of the waste gas returning to the alveoli^5,12,13^. The atelectasis might increase the risk of volutrauma as a constant volume is imparted to a dwindling alveolar volume^1,6^. This frequently occurs during induction of general anesthesia when the fraction of inspired oxygen (FiO_2_) is increased to 100% and it is demonstrated on lung ultrasound and computed tomography (CT) scans of the chest^1,8,9,14–16^. Younger children and infants are especially vulnerable to hypoxemia due to their smaller functional residual capacity (FRC)^4,8,12,13^. Desaturation can subsequently occur by intubation or mask ventilation. Manual ventilation with increased FiO_2_ and the fresh gas flow is then required to recover oxygen saturation (SpO_2_) levels.

Various ventilatory strategies have been proposed to improve gas exchange during general anesthesia^1,6,7,14–16^. Positive end-expiratory pressure (PEEP) is demonstrated as sufficient to minimize atelectasis. It induces only a modest increase in partial pressure of oxygen (PaO_2_)^1,17–19^.

Recruitment maneuver (RM) are ventilation strategies aiming to re-expand atelectatic lung and improve lung compliance^1,9,12^. Recently, a strategy of reopening the atelectatic lung areas during anesthesia with a RM combined with PEEP has been recommended^1,4–6^. It has been shown to improve oxygenation and restore lung volume and may reduce the heterogeneity of V_T_ distribution^1,4–6,20–22^.

However, the change in V_T_ due to RM combined with PEEP after induction of general anesthesia remains unknown. Therefore, we assessed the effects of RM and PEEP on V_T_ and hemodynamics and respiratory function in pediatric patients.

## Methods

### Study design

This prospective observational study was approved by the Ethics Review Board of Kyushu University Hospital (Approval No. 30-446) and was registered with the UMIN Clinical Trials Registry (no. 000050120). The study was conducted from January 2019 to May 2022. The subjects included patients who underwent dental treatment and oral maxillofacial surgery at Kyushu University Hospital under general anesthesia. Patients with upper respiratory tract or preoperative lung disease were excluded from the study. Written informed consent was obtained from the legal guardians of the eligible children before general anesthesia. Pediatric patients (American Society of Anesthesiologists-physical status (ASA-PS): 1–2; aged 3 months–10 years) who underwent general anesthesia for elective dental treatment or oral surgery were divided into three groups: infants (< 1 year), preschool children (1–6 years), and school children (6 > years).

### Anesthesia

Patients were transferred to the operating room without premedication. Anesthesia was then induced by the inhalation of 1–8% sevoflurane after beginning non-invasive monitoring of SpO_2_ by pulse oximetry, electrocardiography (ECG), and non-invasive blood pressure (BP) and heart rate (HR) checks. After loss of consciousness, the sevoflurane concentration was adjusted according to each patient’s hemodynamic condition.

In cases of airway obstruction, airway and jaw thrusts were applied to relieve the obstruction, and ventilation was gently assisted, as necessary. Atropine, fentanyl, and remifentanil were administered after peripheral intravenous access was achieved. Intubation was facilitated with rocuronium, using a Macintosh laryngoscope (Smiths Medical Japan, Tokyo, Japan). The size of the endotracheal tube (ETT) was judged to be appropriate when air leakage was observed at an airway pressure of 10–30 cmH_2_O. When air leakage was either not observed at 30 cmH_2_O or observed below 10 cmH_2_O, the ETT was replaced by another one size above or below^23,24^. The choice of anesthetic maintenance was determined by each anesthesiologist. Pressure control continuous mandatory ventilation (PC-CMV) was performed using a Datex-Ohmeda Aestiva (GE Healthcare, Madison, WI, USA). Anesthesia was maintained using inhalational anesthetics such as sevoflurane, isoflurane, and desflurane, with the administration of fentanyl and remifentanil for analgesia to all patients.

Following tracheal intubation, PC-CMV was initiated with 15 cmH_2_O and a PEEP of 4 cmH_2_O, inspiratory: expiratory ratio of 1:2, and initial ventilatory rate with end-tidal carbon dioxide maintained at 35–45 mmHg by adjusting the respiratory rate (15–20 breaths/min). The FiO_2_ was set to 1.0 throughout the study period and 0.4 during procedures.

### Study intervention

**Figure 1:**
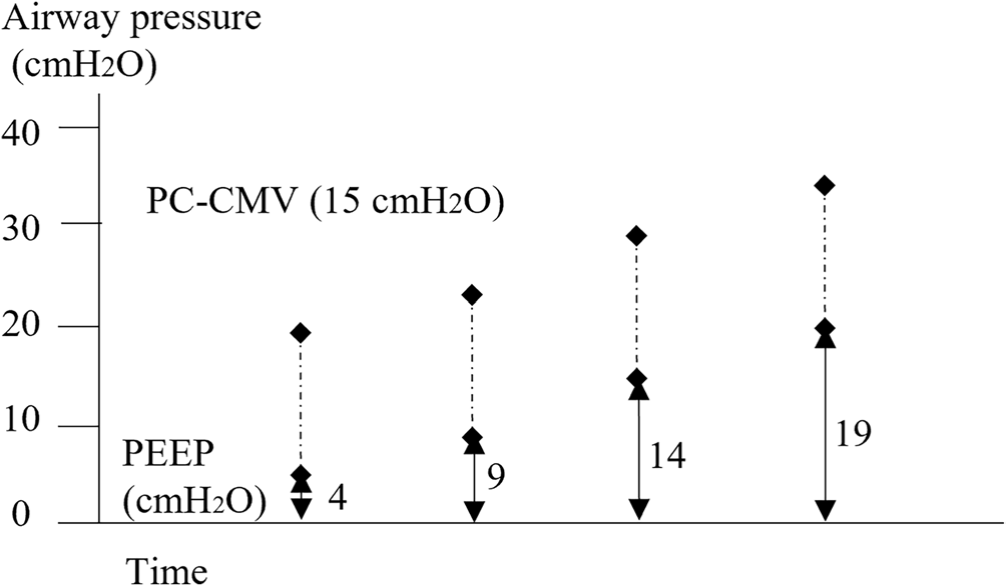
Study protocol. Mechanics of performing the recruitment maneuver (RM) combined with positive end-expiratory pressure (PEEP). The RM was manually performed by pressure control continuous mandatory ventilation with a fixed 15 cmH_2_O driving pressure for 5 s and a step-by-step PEEP increase of 5 cmH_2_O for 3 respiratory cycles up to the target level of 34 cmH_2_O.

The RM was manually performed by PC-CMV with a fixed 15 cmH_2_O driving pressure for 5 s and a step-by-step PEEP increase of 5 cmH_2_O for 3 respiratory cycles up to the target level of 34 cmH_2_O (Fig. 1), followed by maintenance with the previous ventilator settings. This study protocol was stopped immediately if HR and/or SpO_2_ changed by at least 15% from baseline values.

V_T_ at end-expiration at the anesthesia ventilatory monitor, HR, BP and SpO_2_ for 2 min were recorded by an independent observer before and after RM combined with PEEP. The positions of the patients remained unchanged during the measurement period. Following the study protocol, procedure was performed as planned.

### Data analysis

There were no previous studies can be used as a reference. An a priori power analysis was performed using 10 data points based on our clinical experiences. The sample size of 18 patients was calculated based on α = 0.05, standard deviation (SD), and a power of 80% using JMP® 15 (SAS Institute Inc., Cary, NC, USA). Factoring in an estimated dropout rate of approximately 10%, the total sample size for this study was set to 60 patients. The paired t-test was used to compare groups for nonparametric data using MATLAB (version 2015a; MathWorks, Inc., Natick, MA, USA). All values are expressed as the mean ± SD or number (n). The significance level was set at p < 0.05.

### Ethics approval and consent to participate

This prospective observational study protocol was approved by Ethics Review Board of Kyushu University Hospital (Approval No.30-446). Confirming informed consent was obtained from all study participants. Moreover, all methods were carried out in accordance with relevant guidelines and regulations.

## Results

In total, 60 patients were included, with 20 in each group (Table 1). No patients required swapping the ETT. V_T_ at end-expiration before and after RM combined with PEEP were 61.8 ± 9.7 and 80.1 ± 13.9 mL (p < 0.05) in the infant group, 135.8 ± 28.5 and 164.0 ± 36.9 mL (p < 0.05) in the preschool children group, and 217.7 ± 49.7 and 246.9 ± 50.1 mL (p < 0.05) in the school children group, respectively (Table 2). In V_T_ per ideal body weight, similar results were obtained, there were significant differences were observed between before and after RM combined with PEEP in all groups (p < 0.05) (Table 2).

**Table 1:** Patients characteristics.

	Infant group (n = 20)	Preschool children group (n = 20)	School children group (n = 20)
Gender (M/F)	11/9	12/8	14/6
Age (years)	0.4 ± 0.2	3.3 ± 1.3	7.8 ± 1.3
Height (cm)	60.9 ± 13.9	92.8 ± 11.0	122.9 ± 9.0
Body weight (kg)	6.4 ± 0.7	14.0 ± 3.0	23.6 ± 3.9

All values are expressed as mean ± standard deviation (SD) or number (n).

**Table 2:** Variables in the recruitment maneuver (RM) combined with positive end-expiratory pressure (PEEP).

	Before RM combined with PEEP	After RM combined with PEEP	P value
Infant group (n = 20)			
V_T_ (mL)	61.8 ± 9.7	80.1 ± 13.9	< 0.05
V_T_/ideal body weight (mL/kg)	9.7 ± 1.5	12.4 ± 2.4	< 0.05
HR (bpm)	142.3 ± 13.5	144.7 ± 13.3	< 0.05
BP (mmHg)	78.2 ± 12.1/ 41.7 ± 7.0	75.3 ± 10.9 / 38.9 ± 5.1	< 0.05/0.12
Preschool children group (n = 20)			
V_T_ (mL)	135.8 ± 28.5	164.0 ± 36.9	< 0.05
V_T_/ideal body weight (mL/kg)	9.1 ± 1.4	11.7 ± 2.3	< 0.05
HR (bpm)	124.4 ± 15.7	127.2 ± 12.7	0.10
BP (mmHg)	91.3 ± 8.2 / 42.2 ± 9.7	88.3 ± 8.1 / 40.5 ± 7.8	< 0.05/0.12
School children group (n = 20)			
V_T_ (mL)	217.7 ± 49.7	246.9 ± 50.1	< 0.05
V_T_/ideal body weight (mL/kg)	8.5 ± 1.3	10.4 ± 1.7	< 0.05
HR (bpm)	113.3 ± 18.3	117.4 ± 18.4	< 0.05
BP (mmHg)	91.5 ± 6.6 / 46.3 ± 6.7	87.6 ± 8.1 / 43.4 ± 6.6	< 0.05/ < 0.05

A paired t-test was used to compare the groups for nonparametric data.

All values are expressed as mean ± standard deviation (SD).

*V*_*T*_ tidal volume.

Statistical significance was set at p < 0.05.

The HR increased by 2–3% and the BP decreased by 4–7% after RM combined with PEEP in all groups. RM combined with PEEP resulted in an increase in VT per ideal body weight (1.1–1.2%) without respiratory (desaturation, barotrauma) or hemodynamic (hypotension) complications.

## Discussion

In this prospective observational study, we compared V_T_ at end-expiration before and after RM combined with PEEP in mechanically ventilated pediatric patients after the induction of anesthesia. V_T_ after RM combined with PEEP significantly increased (12–18%; 19–29 mL) using PC-CMV with a high driving pressure (15 cmH_2_O) in normal lung, and the V_T_ per ideal body weight was the almost same among the three groups. Furthermore, no hemodynamic or respiratory complications were observed.

Children, most particularly infants, are at an increased risk of developing atelectasis, because of the proximity of the residual volume to the closing volume of the lung and the absence of pathways for collateral ventilation^12,13,21,22^. They have a relatively lower FRC because the highly compliant nature of their chest wall has a reduced ability to counteract the inward elastic recoil of the lung tissue^4,7^. In clinical practice, 100% oxygen has been used as a standard during induction of general anesthesia including tracheal intubation, because it provides the patient enough time to tolerate apnea, it may promote atelectasis formation frequently and immediately (within a few minutes)^5–7,13^. Then, early and active management after induction of anesthesia is need by RM combined with PEEP^1,5,6,8^.

PEEP might be associated with alveolar collapse related to local compression atelectasis caused by overdistended upper lobes^1,6,12^. However, PEEP alone is insufficient in improving oxygenation^5,7,13^. Indeed, PEEP might increase the normally aerated lung fraction along with a reduction in the proportion of poorly aerated lung tissue, although the extent of atelectasis may remain unchanged^5,8^. It is important to maintain PEEP above alveolar closing pressures to prevent de-recruitment following re-expansion of atelectatic lung^4,5^. In contrast, it was reported that PEEP might increase intracranial pressure (IOP), and central venous pressure^9,20,25–27^.

The RM might be an important component of a lung-protective ventilation strategy to re-expand the atelectatic lungs and improve lung compliance^4,7^. High inspiratory pressures (around 40 cmH_2_O) are required to re-open collapsed alveoli, and these must be maintained for a sufficient period of time to allow lung units with slow time constants to re-expand^15,28^.

It was reported that RM combined other levels of PEEP was caused a significant increase in FRC, and effect on the oxygenation for a few hours and lung mechanics in concert with a significant decrease in dead space fraction, although no consensus has been reached on the ideal recruitment strategy^1,18,19,22,28^. This may reduce the amount of pulmonary shunt, despite a concomitant increase in perfusion to poorly ventilated lung units (low VA/Q), which might result in a small but significant reduction in PAO_2_-PaO_2_^1,4–6,18^.

To the best of our knowledge, no study has assessed the effects of V_T_ after RM combined with PEEP. The method of using RM with a stepwise increase in PEEP (4–19 cmH_2_O) sustained inflation of the lungs to a specific peak inspiratory pressure of 34 cmH_2_O based on previous reports^1,6,9,14,20,21^.

We suggest that an increase in V_T_ of about a few ml per body weight was effective in terms of lung compliance and atelectasis. It is also expected to lead to improved respiratory system elastance and dead space, although we did not directly assess on CT or PaO_2_. Pulmonary vascular resistance is greatly increased, or decreased venous return, cardiac output (CO), and HR might occur when ventilating below or above FRC^25^. However, such effects are expected to be transient, with BP and CO returning to the baseline within several minutes, and serious complications may not be common.

Our study had several limitations. First, the use of FiO_2_ 1.0 may have contributed to an increase in the amount of reabsorption atelectasis. However, the use of high FiO_2_ in pediatric anesthetic practice is common; pediatric patients can desaturate rapidly, and respiratory adverse events associated with airway obstruction are frequent, then it was maintained until the beginning of procedures. Second, our patients were graded ASA PS 1–2. The use of RM combined with PEEP in more unstable patients such as acute lung injury was not tested and could increase the side effects.

## Conclusion

The RM combined with a stepwise increase in PEEP significantly increased V_T_ by 12–18% (19–29 mL) without respiratory or hemodynamic complications after the induction of anesthesia. Therefore, this method may safely improve the lung function in pediatric patients.

## Data Availability

The datasets used and/or analyzed during the current study are available from the corresponding author on reasonable request.
